# Students’ psychological distress in an English program embedded in a medical school in a non-English speaking country

**DOI:** 10.5116/ijme.5290.ae54

**Published:** 2013-12-22

**Authors:** Yukari Yamada, Elizabete Loureiro, Miloslav Klugar, Adam Tancred, Katerina Ivanova, Ivana Oborna

**Affiliations:** 1 Faculty of Medicine and Dentistry, Palacky University, Czech Republic; 2 Centre for Medical Education, Faculty of Medicine, University of Porto, Portugal

**Keywords:** Psychological distress, international medical students, social support, stress management

## Abstract

**Objectives:**

To clarify if medical students in an English program in a non-English speaking country are exposed to a higher risk of psychological distress compared to comparable local medical students.

**Methods:**

An online survey was conducted for all medical students both in the English program exclusively for international students (n=235) and in the local program (n=1043) at the Palacky University in the Czech Republic. The Medical Student Well-Being Index (MSWBI) was used to define the student’s psychological distress. Logistic regressions were conducted to find an effect of the study program on the students’ psychological distress, adjusted by age, study year, marital status, residential status, and frequency of contact with significant others, stratified by gender.

**Results:**

44% (n=68) of the respondents in the English program screened positive for psychological distress, and 53% (n=221) in the local program. There was an interaction between gender and program in the association with psychological distress. The higher prevalence of psychological distress in the local program was likely attributed to female students who had frequent contacts with their significant others.

**Conclusions:**

Psychological distress was highly prevalent in a Czech medical school, but there was no overall difference between the international students in the English program and the comparable local medical students.

## Introduction

Psychological distress such as stress, anxiety, depression and burnout has been consistently reported to be prevalent among medical students.^1^^-^^4^ Substantial stresses from the high academic workload and exposure to human suffering in the hierarchical culture of academic medicine have been pointed out, among others, to be underlying causes.^5^^-^^8^ Some personal characteristics, such as perfectionism and the impostor phenomenon,^9^ having a stressful life event^10^ as well as engaging in unhealthy behaviors^11^ were shown to be exacerbating factors of such distress. Psychological distress during medical education deserves serious attention as it was shown to be associated with low academic performance,^12^^,^^13^ cynicism,^14^^,^^15^ an unwillingness to care for the chronically ill^16^ and decreased empathy,^17^ all of which certainly affect the quality of care provided by future physicians.Medical degree programs taught entirely in English have been available in Central and Eastern Europe to attract foreign students since 1990 and in other parts of the world more recently. Students come from all over the world for different reasons. Some might aim at fulfilling their medical education in English and others could not find places to study medicine in their home countries. The graduates would be the so-called international medical graduates (IMGs), unless they remain in the country they obtain the degree from. IMGs are reported to account for a quarter of physicians in the US^18^^,^^19^ and a third in the UK.^20^ It is plausible that those international students studying medicine in a foreign country have to undergo a far tougher workload than other medical students, which would result in more serious psychological problems for them. Ensuring the quality of their education is important if they keep supplying medical workforce in the future. However psychological health of students attending these programs has not been studied yet.Possible relevant literature regarding international students can be found in those dealing with minority /ethnicity of medical students. The international students are certainly the minority in a given society. Although numerous studies have investigated and reported that minority students have disadvantages in academic performance^21^^-^^25^ and that academic performance and psychological status are generally related,^6^^,^^13^^, ^^26^^-^^29^ being minority students does not appear to have a striking negative impact on their psychological status.^9^^,^^30^^-^^33^ Rather some found positive psychological status among minority students, leading to a discussion that life experiences of minorities and stronger social cohesion within their ethnic culture have made them more resilient to difficulties.The difference here is that the international students are less likely to have strong social cohesion within their ethnic culture, as they spend only six years in their country of study.^34^ Being an international student generally means separation from those with whom one has interpersonal relationships. According to the buffering hypothesis of social support, this decreased feeling of belonging would result in decreased immunity to a stressor.^35^ Further, the language required in their daily life and clinical practice is neither English nor their own language.On the basis of these analyses it is likely that the international students in a medical program taught in English in a non-English speaking country might suffer more seriously from psychological distress compared to the other medical students. This study aimed to explore what role the program plays in a student’s psychological distress while taking social support into consideration by comparing the students in the English program with those in the local program at the same university in the Czech Republic.

## Methods

### Study design

Cross-sectional web-based survey was conducted.

### Study setting

The Palacky University in Olomouc, Czech Republic, started a medical program taught in English in 1993 exclusively for foreigners who pay all educational and tuition fees (hereafter, English program). It runs in parallel with the existing medical program taught in Czech (hereafter, local program) for which students do not have to pay. The English program started with a small number of students and has increased the number gradually. It has sent over 200 graduates to over 15 different countries so far.

### Participants

The number of medical students at the end of the year 2012 was 1,278 in total, 1,043 in the local program and 235 in the English program. Fifty eight percent (58%) of the students in the English program were Malaysian, followed by 26% British, and 11% Taiwanese. Other nationalities were represented by fewer than 5% of the students. No statistics about nationality for the local program were available, but they were from either the Czech Republic or its neighboring countries, particularly the Slovak Republic.

### Sample size and sampling methods

All medical students (first through sixth year) were invited to participate in the survey.

### Data collection

All students were sent an e-mail message with a cover letter signed by the vice-dean of the faculty that linked to the online questionnaire in December 2012. Students gave their consent to participate in the survey by starting the online questionnaire. The survey took about 20 minutes to complete. The survey was anonymous to promote honesty in the answers from students. The anonymity was stressed in the cover letter and the questionnaire was carefully made so that authors could never identify any particular student. The study complied with the declaration of Helsinki and was approved by the institutional review board (Etická komise Fakultuní nemocnice Olomouc a Lékařské fakulty UP v Olomouci).A cover letter stated that the purpose of the survey was to better understand the factors that contribute to student well-being and identify how the faculty can make changes to improve student quality of life (QOL). Participants were blinded to any specific hypothesis of the study. The survey included 77 questions regarding demographic information (page1), psychological distress (page2), social support (page3), healthy behaviors (page4), physical activity (page5), academic achievement and the learning environment (page6), and open-ended suggestions for improvement (page7). In this study we used only demographic information and psychological distress since there was a considerable reduction of response rate after the second page among the local students.Demographic information included program (English, local), study-year, gender, age (<25, ≥25 and <30, >30), country of origin (the Czech Republic, the Slovak Republic, Malaysia, Great Britain, others), marital status (married, engaged, non-married relationship or boyfriend/girlfriend, single), frequency of contact with significant others (every day or almost every day, weekly, monthly, less then monthly), and residential status (dormitory, living alone, shared room/apartment, with significant other, with family).Psychological distress was measured by the Medical Student Well-Being Index (MSWBI),^36^ with permission to use from the developers. The MSWBI was developed to identify students in distress, promptly and accurately. It is comprised of seven items covering the domains of burnout (emotional exhaustion and depersonalization), depression, stress, fatigue, and mental and physical QOL. All questions are answered using a simple yes/no. One point is assigned for each ‘yes’ and summary scores on the seven item index range from 0 to 7 (lowest to highest risk for severe distress). Psychometric properties of MSWBI have shown to be good; concurrent validity index of the overall scale is >0.90,^36^ pair-wise percent agreement between raters was >or=85% for clarity, relevance, and representativeness^36^ and Cronbach's alpha was 0.68^36^ and 0.69.^37^ As a threshold score of ≥4, the sensitivity and specificity for identifying students with a low mental QOL or recent suicidal ideation or serious thoughts of dropping out were both ≥90% and the prevalence of a false-negative score (score <4 in students with low mental QOL, suicidal ideation, or serious thoughts of dropping out) was estimated to be 5% to 7%.^38^

### Analysis methods

Demographic variables were dichotomized or trichotomized according to the logical associations and distributions. Study year was trichotomized (1^st^/2^nd^, 3^rd^ /4^th^, and 5^th^ /6^th^). Age was dichotomized (< 25 and ≥ 25) as there were quite a few students who were over 30 years old. Marital status was dichotomized, distinguishing those who were married or engaged from the others. Those who answered as single in the question of marital status were classified as no relation in the frequency of contact. Frequency of contact with significant others was then classified as frequent (every day or almost every day), rare (weekly or less or no answer to this question) or no relation. Residential status was dichotomized distinguishing those living in the dormitory from the others. The MSWBI scores were translated to a dichotomous ‘distressed’ and ‘not distressed’ using the threshold ≥ 4.Differences in basic characteristics and psychological distress by program were evaluated using Pearson chi square tests. Preliminary analyses showed different associations of program with psychological distress between genders: students in the English program were more distressed than students in the local program among male students, while the opposite was seen among female students, thus further analyses were stratified by gender. To see an effect of the program on psychological distress, crude and multivariate logistic regression analyses were performed using STATISTICA version 9. In the logistic regression analyses, model 1 describes the crude association of each variable with students’ distress. Model 2 describes a multivariate analysis with possible confounders that were age, study year and marital status. Model 3 and model 4 added variables that were possible mediators in the association between the program and psychological distress. Model 3 added residential status and model 4 added frequency of contact. The final model included all the variables.Furthermore we made a combined variable between the program and frequency of contact to clarify how frequency of contact with significant others was associated with psychological distress differently between programs.

## Results

A total of 571 students filled out the questionnaire (response rate=45%, 66% in the English program, 40% in the local program). Distribution of respondents’ country of origin in the English program was almost identical with that of all students in the program. The response rate for the local program was lower than that for the English program in every study year, and it was especially low among 5^th^ and 6^th^ year students, which were 24% and 31% respectively.Table 1 showed different characteristics of respondents between programs. More students in the local program were female and younger than in the English program. More international students were single, but also more were married or engaged than the local students, meaning more local students had non-married relationships. More than a half of the local students had contact with their significant others weekly or more, while monthly or less was more common for the international students. The majority of international students lived in the dormitories, while local students had more variety with sharing rooms/apartment as the most frequent option chosen.

**Table 1:** Basic characteristics by program, n=571, Olomouc in the Czech Republic, 2012

Characteristics		Total n (%) N=571	English program n (%) N=154	Local program n (%) N=417	P*
Gender					
	Female	386(68)	91(59)	295(71)	0.008
	Male	185(32)	63(41)	122(29)	
Age					
	<25	519(91)	130(84)	389(93)	0.001
	25-30	46(8)	20(13)	26(6)	
	>30	6(1)	4(3)	2(0)	
Study year					
	1^st^ or 2^nd^	232(41)	49(32)	183(44)	0.004
	3^rd^ or 4^th^	216(38)	59(38)	157(38)	
	5^th^ or 6^th^	123(23)	46(30)	77(19)	
Marital status					
	Married	14(2)	7(5)	7(2)	0.000
	Engaged	20(4)	9(6)	11(3)	
	Non-married relationship/ boyfriend or girlfriend	286(50)	40(26)	246(59)	
	No relation	251(44)	98(64)	153(37)	
Frequency of contact with significant others					
	Not applicable	285(50)	111(71)	174(42)	0.000
	Less than monthly	19(3)	15(10)	4(1)	
	Monthly	17(3)	7(5)	10(2)	
	Weekly	138(24)	6(4)	132(32)	
	Every day or almost everyday	112(20)	15(10)	97(23)	
Residential status					
	Dormitory	222(39)	114(74)	108(26)	0.000
	Living alone	67(12)	13(8)	54(13)	
	With parents/family	92(16)	1(1)	91(22)	
	With significant other	37(6)	3(2)	34(8)	
	Shared room/apartment	153(27)	23(15)	130(32)	

*Pearson chi square tests.

Table 2 showed the results of the MSWBI by programs. Among the multiple domains of psychological distress, low mental QOL and depression were the most prevalent, effecting more than 60% of the students, while fatigue and low physical QOL were less frequent. Nearly a half of the respondents (49%, 283/581) screened positive for psychological distress (MSBWI ≥ 4). In comparison between programs, emotional exhaustion was more prevalent in the English program while fatigue and low physical QOL were more prevalent in the local program.

**Table 2:** Medical Student Well-Being Index (MSWBI) by program, n=571, Olomouc in the Czech Republic, 2012

Well Being Index	Total n (%) N=571	English program n (%) N=154	Local Program n (%) N=417	P*
MSWBI domain				
Burnout-emotional exhaustion	266(47)	86(56)	180(43)	0.007
Burnout –depersonalization	364(64)	86(56)	278(67)	0.170
Depression	356(62)	95(62)	261(63)	0.844
Fatigue	105(18)	14(9)	91(22)	0.000
Stress	302(53)	74(48)	228(55)	0.160
Quality of life –mental	409(72)	107(70)	302(72)	0.490
Quality of life -physical	207(36)	38(25)	169(41)	0.000
Psychological distress (MSWBI>=4)	282(49)	68(44)	221(53)	0,061

*Pearson chi square tests.

Table 3 showed that among women studying in the local program, not living dormitories, and seeing their significant others frequently was associated with a higher probability of psychological distress. The effects of all the three variables on psychological distress, i.e. program, residential status, and frequency of contact, were attenuated when adjusted by each other simultaneously, but frequency of contact remained significant. Table 4 showed that among men the program was not associated with psychological distress both in crude and multivariate associations. Adding frequency of contact in the model (model 4 and final model) did not attenuate the odds ratio of the program among men.

**Table 3:** Crude and multivariate odds ratio (95% CI) for having psychological distress (MSBWI ≥ 4) by program, age, study year, residential status, marital status, and frequency of contact among female students, n=386, Olomouc in the Czech Republic, 2012

Variables	N(cases)	Crude odds	Model1*	Model2^†^	Model3^‡^	Final model^β^
Program						
Local	295(169)	1.00	1.00	1.00	1.00	1.00
English	91(35)	0.47(0.29-0.75)	0.46(0.28-0.76)	0.51(0.29-0.88)	0.55(0.33-0.92)	0.57(0.32-1.01)
Age						
<25	354(185)	1.00	1.00	1.00	1.00	1.00
>=25	32(19)	1.34(0.64-2.79)	1.96(0.86-4.44)	1.99(0.88-4.53)	1.87(0.81-4.31)	1.88(0.81-4.35)
Study year						
1^st^ or 2^nd^	156(85)	1.00	1.00	1.00	1.00	1.00
3^rd^ or 4^th^	140(77)	1.02(0.65-1.61)	1.00(0.63-1.60)	1.00(0.63-1.60)	0.98(0.61-1.57)	0.98(0.61-1.57)
5^th^ or 6^th^	90(42)	0.73(0.43-1.23)	0.69(0.39-1.24)	0.70(0.39-1.25)	0.61(0.33-1.12)	0.61(0.34-1.12)
Marital status						
Married or engaged	17 (8)	1.00	1.00	1.00	1.00	1.00
Others	369(196)	1.27(0.48-3.38)	1.13(0.40-3.18)	1.17(0.41-3.33)	1.39(0.49-3.97)	1.41(0.49-4.04)
Residential status						
Dormitory	137(63)	1.00		1.00		1.00
Others	249(141)	1.53(1.01-2.33)		1.18(0.73-1.92)		1.08(0.66-1.79)
Frequency of contact						
No relation	170(78)	1.00			1.00	1.00
Rare	151(83)	1.44(0.92-2.24)			1.31(0.82-2.10)	1.31(0.82-2.10)
Frequent	65(43)	2.31(1.27-4.18)			2.17(1.13-4.14)	2.13(1.11-4.11)

Logistic regressions: *Model 1 describes multivariate analysis with possible covariates that were age, study year and marital status. ^†^Model 2 added residential status on model 1. ^‡^Model 3 added relationship status on model 2. ^β^Final model included all the variables. Odds ratios with p<0.05 were showed in bold.

**Table 4:** Crude and multivariate odds ratio (95% CI) for having psychological distress (MSBWI≥ 4) by program, age, study year, residential status, marital status, and frequency of contact among male students, n=185, Olomouc in the Czech Republic, 2012

Variables	N (cases)	Crude odds	Model1*	Model2^†^	Model3^‡^	Final model^β^
Program						
Local	122(52)	1.00	1.00	1.00	1.00	1.00
English	63(33)	1.48(0.80-2.73)	1.40(0.74-2.65)	1.55(0.78-3.08)	1.47(0.75-2.87)	1.63(0.80-3.35)
Age						
<25	165(75)	1.00	1.00	1.00	1.00	1.00
>=25	20(10)	1.20(0.47-3.04)	1.11(0.41-3.03)	1.09(0.40-3.00)	1.07(0.39-2.94)	1.04(0.38-2.89)
Study year						
1^st^ or 2^nd^	76(15)	1.00	1.00	1.00	1.00	1.00
3^rd^ or 4^th^	76(40)	1.70(0.90-3.24)	1.63(0.85-3.14)	1.62(0.84-3.14)	1.63(0.83-3.21)	1.63(0.83-3.22)
5^th^ or 6^th^	33(30)	1.28(0.56-2.92)	1.17(0.48-2.85)	1.19(0.49-2.90)	1.17(0.47-2.92)	1.20(0.48-3.01)
Marital status						
Married or engaged	17(8)	1.00	1.00	1.00	1.00	1.00
Others	168(77)	0.95(0.35-2.59)	1.14(0.40-3.20)	1.13(0.40-3.19)	1.25(0.42-3.73)	1.25(0.42-3.74)
Residential status						
Dormitory	85(38)	1.00		1.00		1.00
Others	100(47)	1.10(0.61-1.96)		1.29(0.68-2.45)		1.31(0.69-2.50)
Frequency of contact						
No relation	93(39)	1.00			1.00	1.00
Rare	45(24)	1.58(0.77-3.24)			1.25(0.60-2.58)	1.28(0.61-2.66)
Frequent	47(22)	1.22(0.60-2.47)			1.14(0.51-2.54)	1.12(0.50-2.51)

Logistic regressions: *Model 1 describes multivariate analysis with possible covariates that were age, study year and marital status. ^†^Model 2 added residential status on model 1. ^‡^model 3 added relationship status on model 2. ^β^Final model included all the variables.

Table 5 showed how differently the frequency of contact was associated with psychological distress between programs and genders. There was a strong gradient of association between more frequent contact with significant others and higher psychological distress among the local female students, but not among the international female students.

**Table 5:** Odds ratios (95% CI) for students’ distress (MSBWI ≥ 4) by program and frequency of contact, stratified by gender, n=571, Olomouc in the Czech Republic, 2012

Students	N(case)	Crude	Adjusted*
Male (n=185)			
English + no relation	36(20)	1.00	1.00
English + relation	27(13)	0.74(0.27-2.02)	0.72(0.24-2.20)
Local + no relation	45(16)	0.44(0.18-1.08)	0.44(0.17-1.13)
Local + rare	40(20)	0.80(0.32-1.97)	0.82(0.32-2.11)
Local + frequent	37(16)	0.61(0.24-1.54)	0.51(0.19-1.38)
Female (n=386)			
English + no relation	62(25)	1.00	1.00
English + relation	29(10)	0.78(0.31-1.95)	0.74(0.27-1.99)
Local + no relation	108(53)	1.43(0.76-2.68)	1.29(0.65-2.56)
Local + rare	127(76)	2.20(1.19-4.10)	2.03(1.04-3.99)
Local + frequent	60(40)	2.96(1.41-6.20)	2.98(1.26-6.65)

Logistic regressions: *adjusted by age, study year, residential status and marital status. Odds ratios with p<0.05 were showed in bold.

## Discussion

To our knowledge, this study is the first that has focused on psychological distress of international medical students in a continental European country. There were three main findings. First, approximately half of all students scored 4 or greater in the MSWBI, an indication of distress. Second, despite the special situations faced by the international students, there was no overall difference between international and local students. Third, there was an interaction between gender and program.

### No difference between international and local students in psychological distress-possible interpretations

First, the international students may already have developed strong enough psychological coping styles in order to make them more resilient to study abroad. Apparently, as shown in table1, characteristics of the students were widely different between programs. Although these different characteristics were controlled in the analyses, it is also presumable that students were also at a different risk for psychological distress. The international students may have overcome substantial educational and/or emigrational as well as economic challenges before matriculation. Difficult life experiences might have made them more resilient to overcoming obstacles, just as in the discussion about US minority students.^30^^,^^32^Second, the international students may have other forms of supportive relationships to protect them from psychological distress than do the local students. The fact that the majority of the international students do not have significant others and live in the dorms may imply that their peer student community is stronger than that of local students. Social supports from their peer group may be of special importance to medical students, as it has also been shown for residents.^39^ On the contrary, having frequent contact with significant others had a negative influence on psychological distress, which will be referred to later.Third, among multiple domains of psychological distress, emotional exhaustion, one domain of burnout, may be particularly relevant to the international students; therefore, our findings should be interpreted with caution. The effect of the program on emotional exhaustion remained significant on multivariate analyses and showed that international students had 1.6 times higher risk for having emotional exhaustion with 95% confidence interval of 1.1-2.5 than the local students (data not shown). This finding was inconsistent with nonminority students in the US being more likely to have a higher emotional exhaustion.^30^ Emotional exhaustion appears to be more important than other domains of burnout regarding quality of patient care.^40^

### An interaction between gender and program -impact of frequent contacts with significant others on distress

The higher prevalence of psychological distress among local students compared to the international students was likely to be attributable to the dominant population of local female students who had frequent contacts with their significant others. We hypothesized that international students might be at a higher risk of psychological distress, partly because they have a decreased feeling of belonging. According to the buffering hypothesis of social supportapplicable sources of support for this stressor are close and intimate relationships.^35^ Our findings did not support this theory in our medical students, but was in line with the results of the study by Rospenda et.al, that higher levels of social support outside of the medical school were associated with worse academic performance only among women.^41^ It is understandable that in medical education which places great demands on students' time, close relationships can in turn cause a psychological burden^42^ and there might be a gender difference in risk of distress as a result of role stress from both family demands and medical school demands.^7^ However, it remains unclear why the gender difference was seen only in the local students, not in the international students.

### High prevalence of distress-customized stress management programs are needed

Similar to other studies, we found a high prevalence of psychological distress in medical students regardless of program of study. It was significantly higher than a representative sample in the US medical school using the same scale of psychological distress, which was 31%.^38^ The result highlights the need to promote student well-being.^43^ The first step must be ensuring that all the faculty and hospital members are informed that many of our students are in fact in distress. The second step could aim at introducing proactive programs in order to help students manage their stress effectively.^44^ Stress management intervention could take place in forms of student counseling, support groups, and lectures focusing on stress reduction and coping strategies.^45^^,^^46^ We can suggest here that a stress management program focusing on emotional exhaustion is recommended for the international students. On the contrary, given the negative influence of frequent contact with significant others and the higher prevalence of fatigue and low physical QOL among the local students, a program focusing on skills of how to manage their limited time and to balance important aspects of their lives for professional development and personal fulfillment as a future physician is needed for the local students.^7^ A focus group interview with both local and international students may help to characterize the main sources of stress for students, similar to what was done in a Portuguese study with medical students.^47^

### Limitations

Our study has several limitations. First, it presents results from one medical school at one point in time; therefore the results may not indicate any causality. However, it would not seem unreasonable that the results would generalize to any English track embedded in a medical school in a non-English speaking country. Second, the low response rate, especially among the local students might have caused selection bias and reduction in power. For example, there were only 19 respondents who were over 25 years old and the confidence interval crossed 1.0. It was likely due to the

language used in the questionnaire. We did not translate those established scales to the local language unless valid translations were available. It was intended to avoid any possible changes in nuance and it was indeed a reasonable expectation that local students would not have any problems with clicking one of options to answer questions written in English, but it possibly decreased their motivation to keep filling out the questionnaire. Alternatively the low response rate was caused by a possible association between a person’s psychological distress and the person’s willingness to respond.^30^ Only in a scenario where all the non-respondents were not distressed, our findings would have underestimated a possible adverse effect of the English program. The influence of the special low response rates among the 5^th^ and 6^th^ year of the local students was minimum, as the results were almost identical when the sample was restricted to the students from the 1^st^ to 4^th^ years. Gender difference in response rate was also possible as there were far fewer male respondents in the local program. Lastly, attrition rate was not considered in this study; however, since the rate is usually higher in the local program, there is little concern that we underestimated the difficulty the international students face.

## Conclusion

Psychological distress was highly prevalent in a Czech medical school, but there was no overall difference between the international students in the English program and the comparable local medical students.

## 

### Conflict of Interest

The authors declare that they have no conflict of interest.
